# Endotoxin stabilizes protein arginine methyltransferase 4 (PRMT4) protein triggering death of lung epithelia

**DOI:** 10.1038/s41419-021-04115-7

**Published:** 2021-09-03

**Authors:** Yandong Lai, Xiuying Li, Tiao Li, Toru Nyunoya, Kong Chen, Georgios D. Kitsios, Seyed Mehdi Nouraie, Yingze Zhang, Bryan J. McVerry, Janet S. Lee, Rama K. Mallampalli, Chunbin Zou

**Affiliations:** 1 Division of Pulmonary, Allergy, Critical Care Medicine, Department of Medicine, University of Pittsburgh School of Medicine, Pittsburgh, PA USA; 2 Veterans Affairs Pittsburgh Healthcare System, Pittsburgh, PA USA; 3 Acute Lung Injury Center of Excellence, Division of Pulmonary, Allergy, and Critical Care Medicine, University of Pittsburgh, Pittsburgh, PA USA; 4 Department of Environmental and Occupational Health, Graduate School of Public Health, University of Pittsburgh, Pittsburgh, PA USA; 5 Department of Medicine, Ohio State University College of Medicine, Columbus, OH USA

**Keywords:** Ligases, Epigenetics, Ubiquitin ligases, Infection

## Abstract

Lung epithelial cell death is a prominent feature of acute lung injury and acute respiratory distress syndrome (ALI/ARDS), which results from severe pulmonary infection leading to respiratory failure. Multiple mechanisms are believed to contribute to the death of epithelia; however, limited data propose a role for epigenetic modifiers. In this study, we report that a chromatin modulator protein arginine *N*-methyltransferase 4/coactivator-associated arginine methyltransferase 1 (PRMT4/CARM1) is elevated in human lung tissues with pneumonia and in experimental lung injury models. Here PRMT4 is normally targeted for its degradation by an E3 ubiquitin ligase, SCF^FBXO9^, that interacts with PRMT4 via a phosphodegron to ubiquitinate the chromatin modulator at K228 leading to its proteasomal degradation. Bacterial-derived endotoxin reduced levels of SCF^FBXO9^ thus increasing PRMT4 cellular concentrations linked to epithelial cell death. Elevated PRMT4 protein caused substantial epithelial cell death via caspase 3-mediated cell death signaling, and depletion of PRMT4 abolished LPS-mediated epithelial cell death both in cellular and murine injury models. These findings implicate a unique molecular interaction between SCF^FBXO9^ and PRMT4 and its regulation by endotoxin that impacts the life span of lung epithelia, which may play a key role in the pathobiology of tissue injury observed during critical respiratory illness.

## Introduction

Acute lung injury and acute respiratory distress syndrome (ALI/ARDS) is a major public health concern with high mortality of approximately 30–40% [1–5]. Pathologically, ALI/ARDS is characterized by lung inflammatory cell infiltration, and alveolar-capillary leak of edema fluid into the airways leading to hypoxia and respiratory failure. Viral and bacterial lower-airway infection is a leading cause of ALI/ARDS, which contributes to approximately 40% of the total ALI/ARDS patients [6–9]. ALI/ARDS is also observed during pandemic coronavirus infection, caused by severe acute respiratory syndrome coronavirus 2 [10–13]. Among the mechanisms of the ALI/ARDS pathogenesis, lung epithelial cell death is a crucial signature event linked to loss of integrity of the lung lining leading to an alveolar–capillary leak with edema. Although many pathways have been described causing epithelial cell death [5, 14], there is limited investigation of unique molecular pathways involving epigenetic mechanisms. Molecular mechanistic studies are of particular importance in uncovering potential effective targets for therapeutic intervention in this illness.

Protein arginine methyltransferase 4 (PRMT4/CARM1) governs a range of life processes including gene transcription, proliferation, RNA splicing, development, nuclear factor (NF)-κB mediated inflammation, and p53-related signal transduction via its epigenetic and non-epigenetic functions [15–21]. PRMT4 is crucial in development, as knockout of PRMT4 in mice leads to neonatal death and developmental defects in respiratory system. In these mice, alveolar type II epithelial cells that are crucial for surfactant production and ion transport are smaller than normal but with increased numbers. Notably, depletion of PRMT4 blocks type II alveolar cell differentiation into type I cells, and no type I alveolar epithelial cells are generated in PRMT4 knockout mice lung [22]. Aberrant expression of PRMT4 proteins is identified in a number of neoplastic tissues that regulates many aspects of cancer, including carcinogenesis, metastasis, recurrence, and drug resistance [23, 24]. PRMT family members also regulate apoptosis as PRMT2 inhibits NF-κB signaling in mouse embryo fibroblasts [25]. PRMT4 expression induced by high-glucose loading triggers apoptosis of human retinal pigment epithelial cells via H3R17 di-methylation [26]. Recent studies reported that specific expression of PRMT4 in adipose tissue promotes lipolysis and increases serum triglycerides in type 1 diabetic mice [27]. Cardiac-specific expression of PRMT4 promotes apoptosis of cardiomyocytes in mouse models [28]. However, how bacterial pathogens regulate PRMT4 at the posttranslational level is not fully understood.

The ubiquitin proteasome system degrades most cellular proteins in living cells and at times in coordination with the gene transcriptional machinery. Ubiquitination proceeds in an enzymatic cascade, in which the final step involves an E3 ubiquitin ligase that recognizes a protein substrate to catalyze ligation of ubiquitin to the substrate. This process often results in substrate disposal within the proteasome. Among E3 ubiquitin ligases, a family of SCF (Skp1-Cullin1-Fbox) E3 proteins have been linked to bacterial infection, antiviral immunity, and host defense [29–36]. Interestingly, the F-box protein FBXO9, a component of SCF^FBXO9^ E3 ligase complex, was identified in a pooled RNAi screen as a lipopolysaccharide (LPS)-responsive protein in the context of interleukin (IL)-6 production to regulate innate immunity [37]. FBXO9 may be associated with patient survival in multiple myeloma, is linked to adipocyte development, and targets peroxisome proliferator-activated receptor gamma (PPARγ) for degradation [38–41]. Aside from these studies, our understanding of this E3 ligase subunit is limited, particularly as it relates to sepsis.

In this study, we identified that microbial factors (i.e., endotoxin) increase PRMT4 expression by extending its cellular protein life span thereby inducing lung epithelial cell death in experimental lung injury models. Our data show that LPS co-ordinately downregulates the expression of an E3 ubiquitin ligase SCF^FBXO9^ that normally targets PRMT4 for its elimination in cells. Hence, the molecular balance between SCF^FBXO9^ and PRMT4 is modulated during infection that alters viability of lung epithelia.

## Results

### Gram-negative bacterial-derived endotoxin increases PRMT4 protein expression

**Fig. 1:**
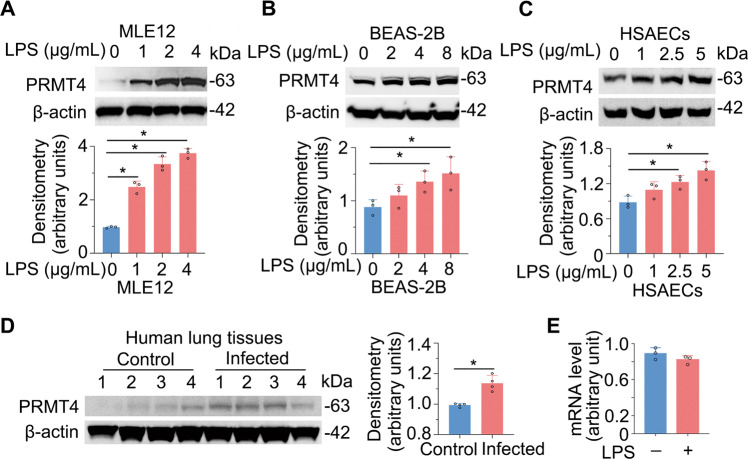
LPS increases PRMT4 protein in epithelia. **A** MLE12 cells were treated with LPS as indicated; cell lysates were immunoblotted with PRMT4 and β-actin antibodies. **B** BEAS-2B cells were treated with LPS as indicated; cell lysates were immunoblotted with PRMT4 and β-actin antibodies. **C** Primary human small airway epithelial cells (HSAECs) were treated with LPS as indicated; cell lysate was collected and analyzed with PRMT4 and β-actin antibodies. For **A**–**C**, relative expression of PRMT4 was plotted in the below panels. **D** Lung tissue lysates from deidentified human lung samples from normal and infected patients were analyzed for PRMT4 and β-actin by immunoblotting. Densitometry was presented in the right panel. **E** *PRMT4* mRNA levels were determined by qRT-PCR in LPS-treated bronchial epithelial cells. Experiments *n* = 3. Statistical significance was indicated with asterisk “*” between groups where *P* < 0.05 vs. control (0).

We identified that *Escherichia coli*-derived LPS increases PRMT4 protein levels in lung alveolar epithelial type II-like MLE12 cells (Fig. 1A). LPS-mediated PRMT4 accumulation was observed in a concentration-dependent manner in the cells. We also observed that LPS increased PRMT4 cellular concentrations in human lung epithelial BEAS-2B cells (Fig. 1B) and in primary human small airway epithelial cells (HSAECs; Fig. 1C). To assess clinical relevance, we investigated PRMT4 protein levels in de-identified human lung tissue samples [42]. Immunoblotting results showed that protein levels were higher in infected lung tissue samples (Fig. 1D). We then assayed *PRMT4* mRNA levels using quantitative reverse transcription–polymerase chain reaction (qRT-PCR), with results showing no remarkable differences in LPS-treated and untreated BEAS-2B cells (Fig. 1E). These results suggest that bacteria elevate PRMT4 protein levels possibly by impairing its protein degradation.

### PRMT4 is a labile protein degraded via the ubiquitin proteasomal machinery

**Fig. 2:**
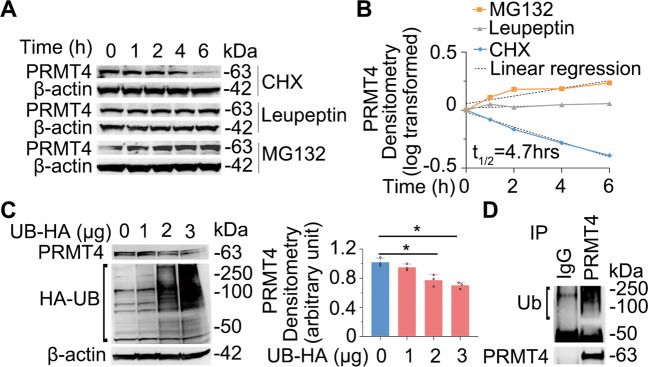
PRMT4 is a labile protein degraded via the ubiquitin proteasomal machinery. **A** BEAS-2B cells were treated with cycloheximide (CHX), leupeptin, or MG132 as indicated. Cell lysates were immunoblotted with PRMT4 and β-actin antibodies. **B** Densitometry results in **A** were analyzed in semi-log format with GraphPad prism 5 and the results were plotted. **C** Ectopic expression of ubiquitin reduces PRMT4 protein in a concentration-dependent manner. Densitometry was plotted in the right panel. **D** PRMT4 is polyubiquitinated as shown by co-immunoprecipitation (Co-IP) of precipitates analyzed with ubiquitin and PRMT4 antibodies.

The availability of a specific proteins in cells is the result of dynamic homeostasis between its gene transcription, translational efficiency, and protein decay. However, the molecular mechanism(s) of PRMT4 protein degradation is not fully elucidated [43]. Immunoblotting results confirmed that the half-life of PRMT4 protein is approximately 4.7 h in human lung epithelial BEAS-2B cells (Fig. 2A, B). PRMT4 degradation was via the proteasome but not the lysosome, as the proteasome inhibitor MG132 but not the lysosome inhibitor leupeptin accumulated PRMT4. Proteins degraded via the proteasome are generally poly-ubiquitinated and ubiquitin could be a limiting factor for protein degradation in a specified cellular compartment as the distribution of ubiquitin can be spatially uneven. Ectopic expression of ubiquitin accelerated PRMT4 turnover in a ubiquitin-dependent manner (Fig. 2C). Results from immunoprecipitation (IP) studies showed that PRMT4 was also poly-ubiquitinated in BEAS-2B cells (Fig. 2D). In all, these data indicate that PRMT4 is a labile protein with a half-life of about 4.7 h degraded via a ubiquitin proteasomal machinery.

### SCF^FBXO9^ E3 ubiquitin ligase targets PRMT4 in lung epithelial cells

**Fig. 3:**
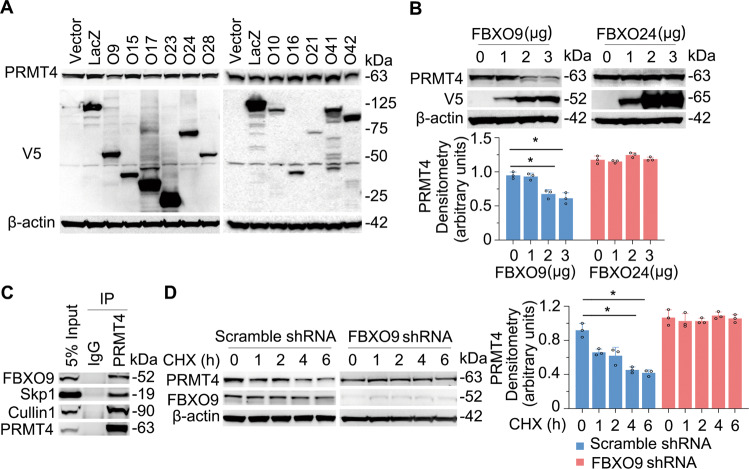
SCF^FBXO9^ E3 ubiquitin ligase specifically degrades PRMT4 in lung epithelia. **A** Screen of F-box (FBXO) family members revealed that overexpression of *FBXO9* selectively degrades PRMT4 in BEAS-2B cells. **B** Ectopically expressed *FBXO9* plasmid degrades PRMT4 in a concentration-dependent manner not observed with *FBXO24*. Densitometric results are presented in the bottom panels. **C** Co-IP studies showing that PRMT4 interacts with Skp1 and Cullin 1, components of SCF E3 ligase. **D** Knockdown of *FBXO9* stabilizes PRMT4 using protein half-life studies with cellular exposure to cycloheximide (CHX) for various times to inhibit protein synthesis. The relative expression of PRMT4 was plotted in the right panel. *P < 0.05 vs. time 0. Experiments *n* = 3.

Protein ubiquitination is a cascade involving E1, E2, and E3 enzymes, in which an E3 ubiquitin ligase is substrate specific. By screening a library of SCF-E3 ubiquitin ligases, we identified that SCF^FBXO9^ specifically degrades PRMT4 (Fig. 3A). Further, PRMT4 protein was degraded in an FBXO9 plasmid concentration-dependent manner (Fig. 3B, left panels). As a control, FBXO24, another F-box protein, did not degrade PRMT4 (Fig. 3B, right panels). IP studies showed that FBXO9 protein associates with PRMT4, as do the SCF complex components Skp1 and Cullin1 (Fig. 3C). Moreover, knockdown of *FBXO9* with small hairpin RNA (shRNA) stabilized PRMT4 as compared with that of a scrambled shRNA control (Fig. 3D). Overall, these data indicate that PRMT4 is specifically targeted for its cellular disposal by the SCF^FBXO9^ ubiquitin proteasomal machinery.

### SCF^FBXO9^ ubiquitinates PRMT4 at K228

**Fig. 4:**
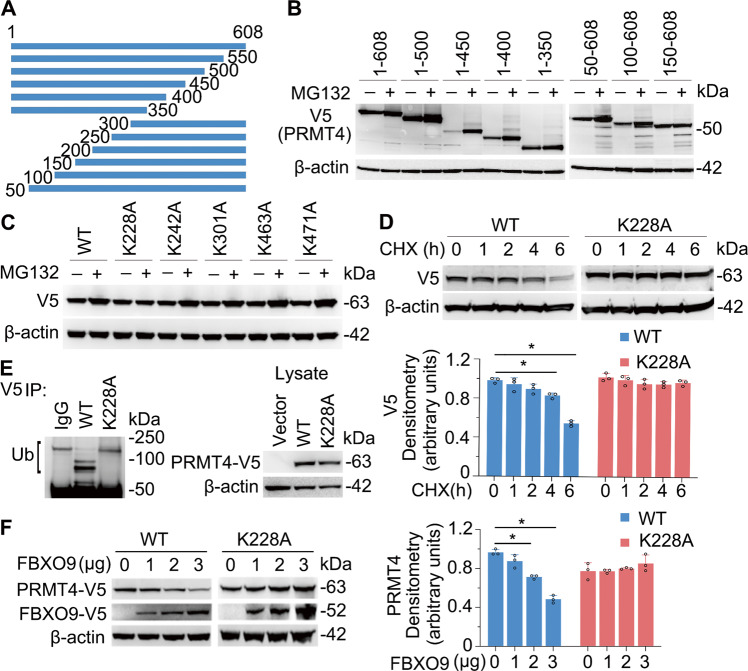
SCF^FBXO9^ ubiquitinates PRMT4 at K228. **A** Schematic presentation of PRMT4 truncation constructs that were expressed in BEAS-2B cells. **B** Fragment 150–608 does not accumulate after MG132 treatment. **C** Cellular expression of a K228A point mutant does not accumulate after MG132 treatment. **D** Cellular expression of a K228 mutant of PRMT4 is stable after CHX treatment. **E** Cellular expression of a V5-PRMT4 K228A mutant vs. a V5 wild-type (WT) PRMT4 is not ubiquitinated. Constructs were expressed in cells and immunoprecipitated using V5 antibodies and probed with ubiquitin antibodies. The right blot shows the levels of WT and mutant construct protein expression in cells. **F** Ectopic expression of *FBXO9* does not degrade the PRMT4 K228A mutant. *P < 0.05 vs. “0.” Experiments *n* = 3.

We next fine mapped the ubiquitination site(s) within PRMT4. We generated and expressed truncated *PRMT4* constructs in cells to examine protein degradation of the truncations (Fig. 4A). Cellular expression of constructs harboring carboxyl terminal deletions resulted in PRMT4 accumulation suggesting that molecular signatures that mediate protein disposal are not localized within these fragments (Fig. 4B, left panels). We next examined NH_2_-terminal deletions and observed that fragments 50–608 and 100–608 also resulted in PRMT4 accumulation after MG132 treatment. However, fragment 150–608 prevented PRMT4 accumulation (Fig. 4B, right panels), suggesting that aa100–150 contains a degradation element. We analyzed a series of lysine residues resided in the truncation mutant containing aa100–608. As compared with that of wild-type (WT) PRMT4 and other mutants, a K228A mutant was stable from proteasomal degradation (Fig. 4C, D). The K228A mutant was less ubiquitinated as compared with that of WT PRMT4 (Fig. 4E, left panels), which indicated that K228 is a ubiquitin acceptor site. In addition, mutation of K228 abrogated FBXO9-mediated PRMT4 degradation (Fig. 4F). These data suggest that K228 may be one authentic SCF^FBXO9^-catalyzed ubiquitin acceptor site.

### SCF^FBXO9^ recognizes a PRMT4 phosphodegron

**Fig. 5:**
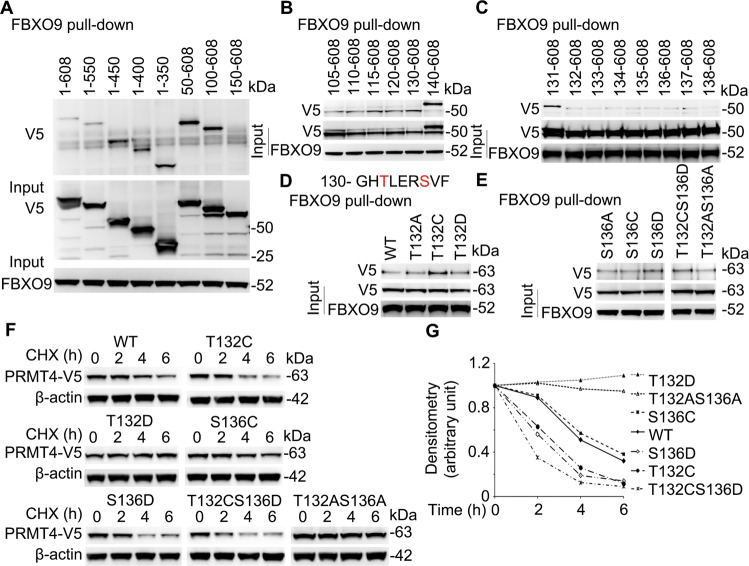
FBXO9 docks on a phosphodegron 132TxxxS within PRMT4. **A** BEAS-2B cells were ectopically expressed with various deletion V5-*PRMT4* plasmid constructs and lysates subjected to FBXO9 immunoprecipitation from MLE12 cell lysates and subsequent V5 immunoblotting to assess FBXO9:V5 interaction. Pull-down assays suggest that aa100–150 of PRMT4 is important for FBXO9 binding. **B**, **C** Fine mapping the FBXO9-binding sites with pull-down assays indicate that a phosphodegron 132TxxxS is critical for FBXO9 binding. **D**, **E** Pull-down assays with phosphorylation/dephosphorylation mimics indicate that T132 dephosphorylation and S136 phosphorylation promote the degron binding to FBXO9. **F** T132 dephosphorylation and S136 phosphorylation accelerate PRMT4 degradation using cycloheximide (CHX). **G** Densitometric results of **F** were plotted. Experiments *n* = 3.

As SCF E3 ubiquitin ligases specifically interact with their substrates, we next studied the FBXO9 docking site within PRMT4. We focused on the NH_2_-terminus because the above studies suggested that aa100–150 contains a degradation element, possibly an E3 ligase docking site. Results from in vitro pull-down assays showed that deletion of NH_2_-terminal 150 aa impairs PRMT4 and FBXO9 binding (Fig. 5A). We further fine mapped the docking site for FBXO9 within PRMT4 by pull-down assays (Fig. 5B, C). Pull-down results showed that amino acids after aa131 were critical for FBXO9 binding. We analyzed the sequence and identified a phosphorylation motif in this region: 132-TxxxS. To test whether this motif is a potential phosphodegron, we substituted T132 and S136 with a cysteine to mimic dephosphorylation or introduced an aspartic acid at T132 to mimic a phosphorylation site. Pull-down results suggest that a dephosphorylated T132 and phosphorylated S136 sites constituted an optimal FBXO9-binding motif constituting a phosphodegron for E3 SCF ligase interaction (Fig. 5D, E). Consistent with these observations, our recent published study showed that glycogen synthase kinase-3 beta (GSK-3β)-mediated T132 phosphorylation stabilized PRMT4 [43]. We further tested the protein stability with these mutations (Fig. 5F, G). T132C and S136D mutants degraded faster than that of WT PRMT4. A S136C mutant displayed greater protein stability. T132D and T132AS136A double mutants completely stabilized the protein from degradation. However, a T132CS136D PRMT4 variant when expressed in cells degraded most rapidly (Fig. 5F, G). In all, these data indicate that SCF^FBXO9^ engages a phosphodegron 132-T^d^xxxS^p^ to ubiquitinate PRMT4 at K228.

### PRMT4 promotes caspase 3 activation and cell death in lung epithelial cells

**Fig. 6:**
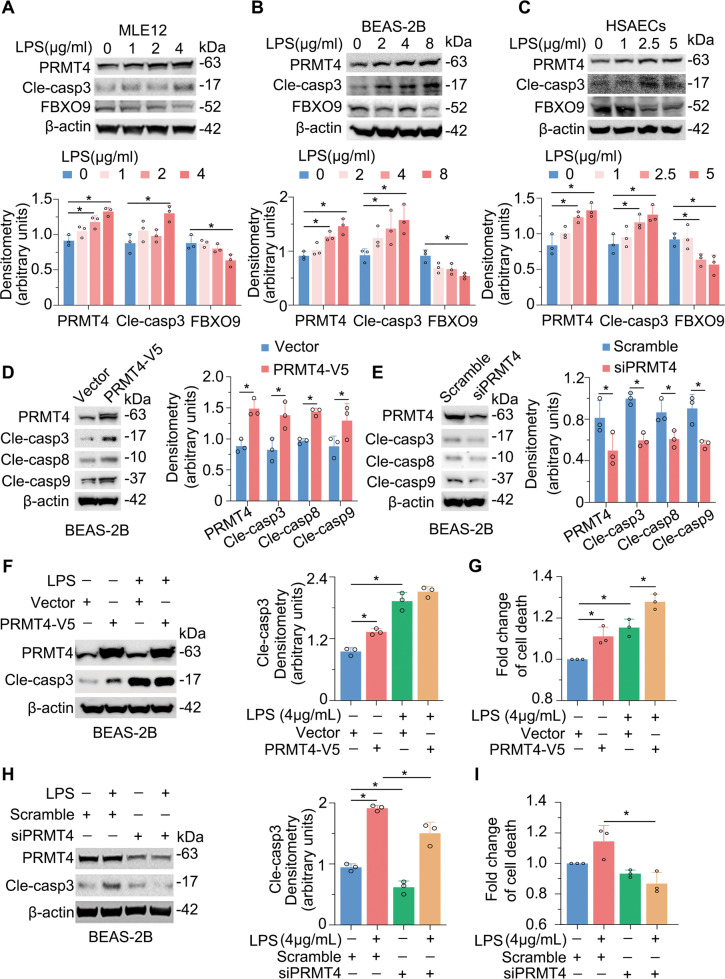
Endotoxin reduces FBXO9 levels leading to PRMT4 accumulation and caspase 3 activation in lung epithelia. **A**–**C** MLE12 cells, BEAS-2B cells, and human primary small airway epithelial cells (HSAECs) were treated with LPS as indicated. Cell lysates were analyzed by PRMT4, cleaved caspase 3, FBXO9, and β-actin immunoblotting. Shown below is densitometric analysis of immunoblots. **D** Overexpression of *PRMT4* increases cleaved caspase 3, 8, and 9 baseline levels in BEAS-2B lung epithelial cells. **E** Knockdown of *PRMT4* in BEAS-2B epithelial cells with shRNA reduces cleaved caspase 3, 8, and 9 expression. **F**, **G** Overexpression of *PRMT4* enhances LPS-induced caspase 3 activation (**F**) and causes BEAS-2B lung epithelial cell death. Cell death is determined using LDH assay and the data are normalized with that from untreated control cells. **H**, **I** Silencing of *PRMT4* inhibits LPS-induced caspase 3 activation (**H**) and cell death (**I**) in BEAS-2B cells. *P < 0.05. Experiments *n* = 3.

We next assessed a potential pathophysiological role for elevated PRMT4 after bacterial infection. We tested whether actions of LPS on lung epithelial cell death are mediated by PRMT4 through its E3 ligase, SCF^FBXO9^. We studied cell death using activated caspase 3 as a marker in a variety of cell types including mouse type II MLE12 cells, human airway epithelial BEAS-2B cells, and human primary small airway epithelial cells after 8 h of LPS stimulation. As predicted, caspase 3 was activated in all of these cells, and its activation occurred in an LPS concentration-dependent manner (Fig. 6A-C, second upper panels). Notably, LPS downregulated FBXO9 protein variably in all the tested cells, suggesting a mechanism whereby LPS stabilizes PRMT4 in cells via depletion of SCF^FBXO9^ E3 ubiquitin ligase (Fig. 6A–C, second to lowest panels). To understand the potential role(s) of PRMT4 in caspase cleavage in lung epithelial cells, we ectopically expressed *PRMT4* in BEAS-2B cells. Immunoblotting results showed that *PRMT4* ectopic expression was sufficient to activate caspase 3, 8, and 9 in BEAS-2B cells (Fig. 6D). We then depleted *PRMT4* expression using small interfering RNA (siRNA) to further examine our findings. Immunoblotting results showed that depletion of *PRMT4* by siRNA reduced caspase 3, 8, and 9 activation to lower levels compared to the scrambled control (Fig. 6E). Ectopic expression of *PRMT4* promoted LPS-induced caspase 3 activation (Fig. 6F) as well as epithelial cell death (Fig. 6G). In contrast, depletion of *PRMT4* blocked LPS-mediated caspase 3 activation to baseline levels (Fig. 6H) and cell death of epithelia (Fig. 6I). Thus, these data suggest that LPS increases PRMT4 protein level possibly via downregulation of FBXO9, thereby facilitating the methyltransferase to trigger caspase 3 signaling in lung epithelial cells.

### PRMT4 mediates lung epithelial cell death in experimental ALI

**Fig. 7:**
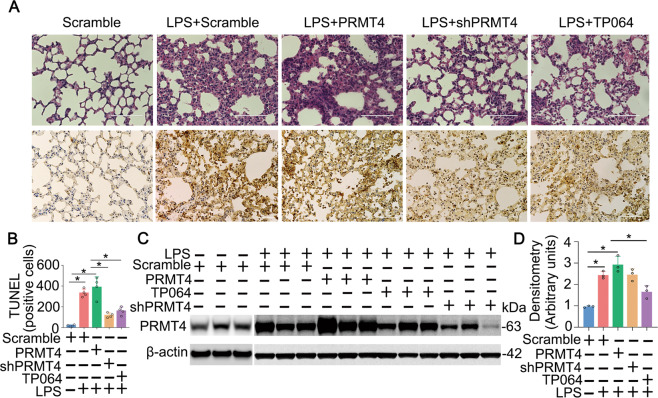
Inhibition of PRMT4 suppresses cell death in a LPS lung injury model. **A**–**D** PRMT4 was silenced or overexpressed by i.t. administered lentiviral constructs for 14 days. Mice were then given (i.t.) LPS with or without a PRMT4 inhibitor as indicated for 24 h (*n* = 8). Lung tissues were stained with hematoxylin–eosin (H&E) (**A**, upper panels) and TUNEL staining (**A**, lower panels). **B** TUNEL-positive cells in lung tissues were quantitated. **C** *PRMT4* overexpression and knockdown in lung tissues from **A** were analyzed by immunoblotting. The data from **C** were quantitated and plotted in **D** (*n* = 3). *P < 0.05 vs. scrambled control. Scale bar = 100 μm.

To test our hypothesis biologically, we assessed PRMT4 in an LPS lung injury mouse model. We overexpressed *PRMT4* or knocked down *PRMT4* using lentiviral constructs (1 × 10^7^ colony-forming units/mouse, intratracheal [i.t.] administration) in mice (C57BL/6J) for 14 days. LPS (5 mg/kg, i.t. in phosphate-buffered saline (PBS) buffer) was administrated (i.t.) into mice for 24 h. Results from histological studies showed that administration of LPS caused lung inflammation with inflammatory cell infiltration. Overexpression of *PRMT4* enhanced lung inflammation and knockdown of *PRMT4* or application of PRMT4 inhibitor TP064 (10 μg/kg) attenuated lung inflammation (Fig. 7A, upper panels). Results from terminal deoxynucleotidyl transferase-mediated dUTP-fluorescein nick end labeling (TUNEL) staining showed that LPS caused cell death in lung tissues, in which overexpression of *PRMT4* enhanced cell death and knockdown of *PRMT4* or application of PRMT4 inhibitor attenuated cell death in the lung (Fig. 7A, lower panels, and Fig. 7B). Immunoblotting results from lung indicated that i.t. administration of LPS or lentiviral particles did modulate PRMT4 protein levels in lung tissues (Fig. 7C, D). A PRMT4-specific inhibitor TP064 partially protected lung epithelial cells from death. Overall, these data suggest that LPS-enhanced PRMT4 protein expression promotes lung epithelial cell death and targeting PRMT4 attenuates severity of cellular injury in an experimental mouse lung inflammation model.

## Discussion

**Fig. 8:**
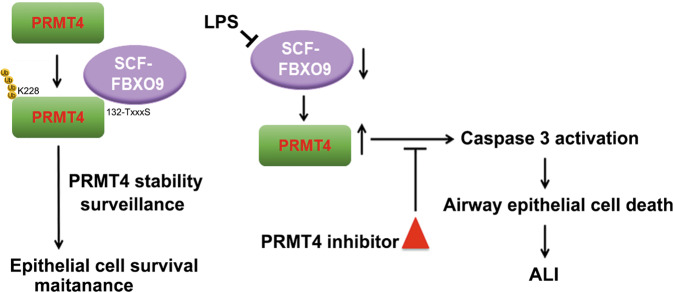
Schematic presentation of the role of PRMT4 and SCF^FBXO9^ in airway cell death. Left: PRMT4 is normally degraded via a SCF^FBXO9^ E3 ligase-mediated ubiquitin proteasomal pathway to maintain airway epithelial cell survival. SCF^FBXO9^ recognizes PRMT4 via a phosphodegron and poly-ubiquitinates PRMT4 at K228. Bacterial endotoxin reduces FBXO9 levels that results in PRMT4 protein accumulation. Increased PRMT4 activates caspase 3 to induce lung epithelial apoptosis during bacterial infection.

Lung epithelial cell death is one of the mechanistic centerpieces of ALI/ARDS, a disorder resulting from severe pulmonary infection underscoring an unmet need to identify new molecular targets for therapeutic intervention. The fundamentally new findings in this study are that (i) PRMT4 protein is elevated in infected lung tissue of human subjects and lung epithelial cells exposed to bacterial endotoxin; (ii) SCF^FBXO9^, via unique molecular signatures, normally targets the labile protein PRMT4 for its cellular elimination, but the E3 ligase is decreased in cells by endotoxin resulting in stabilized PRMT4 that mediates lung epithelial cell death; and (iii) genetic depletion or chemical inhibition of PRMT4 attenuates lung epithelial cell death and protects mice after endotoxin-induced lung injury (Fig. 8). Dysregulation of PRMT4 has been reported in many pathophysiological settings. PRMT4 is aberrantly expressed in breast, prostate, and colorectal cancers that is associated with a poor prognosis by promoting tumor progress and cancer metastasis [24, 44–49]. Consistent with these observations, we identified that PRMT4 expression is increased in ALI. Here the proinflammatory PRMT4 protein was upregulated in cellular and mouse injury models and in human infected lung tissue samples. Increased PRMT4 protein was both required and sufficient to cause lung epithelial cell death after endotoxin exposure and this finding may be one mechanism underlying the pathogenesis of ALI [50].

A number of SCF-E3 ubiquitin ligases or deubiquitinases are found to regulate protein stability and availability of key molecules in the pathogenesis of ALI/ARDS. SCF^FBXL2^ regulates the protein stability of TRAF proteins in viral infection; mutations in FBXL2 may impair TRAF protein stability that augments inflammatory response [25, 51]. SCF^FBXL19^ specifically interacts with ST2L receptor for IL-33 that abrogates the proapoptotic and inflammatory effects of IL-33 in ALI models [23]. In previous work, we identified that SCF^FBXL18^ turns over a chromatin modulator Mortality factor 4 like 1 (MORF4L1) to regulate the life span of lung epithelial cells. The Gram-negative bacterium *Pseudomonas aeruginosa* disrupts the interaction between FBXL18 and MORF4L1 that elevates cellular MORF4L1 protein levels to cause lung epithelial cell death [42]. FBXO9 is a less studied E3 ubiquitin ligase with functions in muscle and neuronal development [52, 53]. Aberrant expression of FBXO9 to regulate mammalian target of rapamycin signaling by targeting Tel2 and Tti1 degradation promotes survival of multiple myeloma cells [38]. FBXO9 has been shown to be related to innate immunity by responding to LPS stimulation in mouse models [37]. FBXO9 interacts with PPARγ to ubiquitinate this receptor for proteasome degradation [41]. PPARγ is an important player in the immune system for maturation and function of various immune system-related cell types, such as monocytes/microphages, dendritic cells, and lymphocytes [54].

Here we identified a new molecular target of FBXO9 in its control of a protein arginine methyltransferase, PRMT4, that participates in ALI pathogenesis through control of lung epithelial cell viability. Endotoxin LPS decreases FBXO9 that impairs degradation of PRMT4 protein. Elevated PRMT4 activates both intrinsic and external apoptotic pathways to cause epithelia death. Before PRMT4 is targeted for ubiquitination and subsequent degradation, PRMT4 is recognized by the E3 ubiquitin ligase SCF^FBXO9^ through a phosphodegron. In this motif (132-T^d^xxxS^p^), the phosphorylation status of serine and threonine residues appear to be differentially modified for optimal FBXO9 binding and subsequent ubiquitination. Phosphodegrons have been widely cited in governing the stability of crucial molecules in the pathogenesis of diseases. In this regard, we previously identified that oxidative stress reduces PRMT4 levels via downregulation of a protein kinase GSK-3β. GSK-3β phosphorylates T132 that is crucial in regulating the affinity between the phosphodegron and the E3 ubiquitin ligase [43]. Interestingly, in the present study, we observed that LPS downregulates the E3 ubiquitin ligase component FBXO9 at the protein level to stabilize PRMT4 protein. Depletion of *PRMT4* by *shRNA* or with a PRMT4 small-molecule inhibitor improved mouse lung cell viability in an LPS lung injury mouse model, suggesting that lung epithelial cell death mediated by this protein arginine methyltransferase may be a critical factor underlying the pathogenesis of endotoxin-mediated injury. Thus, it is reasonable to postulate that there exists a delicate balance between SCF^FBXO9^ and PRMT4 that, in part, governs the life span of epithelia during endotoxin stress. The results suggest a potential for pharmaceutical targeting of PRMT4 to reduce the severity of tissue injury in ALI/ARDS.

## Materials and methods

### Cell lines and reagents

Human lung epithelial BEAS-2B cells and mouse alveolar epithelial MLE12 cells were from ATCC, maintained with HITES medium (Gibco) containing 10% fetal bovine serum. Cells were maintained in a 37 °C incubator in the presence of 5% CO_2_. Human primary small airway epithelial cells were from ATCC and cultured with airway cell basal medium (Cat#: ATCC PCS-300-040) accompanied with bronchial epithelial growth kit (Cat#: ATCC PCS-300-040). V5 antibody (Cat#: 37–7500), pcDNA3.1D-His-V5-TOPO cloning kit (Cat#: K490001) and *E. coli* Top10 competent cells (Cat#: C404006) were from Invitrogen (St. Louis, MO). PRMT4 (Cat#:12495), cleaved caspase 3 (Cat#: 9661), cleaved caspase 8 (Cat#: 9748), and cleaved caspase 9 (Cat#: 9505) antibodies were from Cell Signaling (Danvers, MA). Ubiquitin (Cat#: sc-166553, lot: A2710) antibody was from Santa Cruz Biotechnology (Santa Cruz, CA). FBXO9 (Cat#: PA5-23474) antibody was from Thermo Fisher (Rockford, IL). The PRMT4 shRNA was from Origene (Rockville, MD). Cycloheximide (Cat#: ALX-380-269-G001, lot: 01061518) and Ubiquitin aldehyde (Cat#: BML-UW8450-0050, lot: 07021447) were from Enzo Life Sciences (Farmingdale, NY). β-Actin (Cat#: A3853) antibody and bacterial lipopolysaccharide (LPS) from *E. coli* O111:B4 (Cat#: L4391, lot: 115M4090V) were from Sigma (Carlsbad, CA). MG132 (Cat#: F1101, lot: F11052079) was from UBPBio (Aurora, CO). TP064 (Cat#: 6008) was from Tocris Bioscience (Ellisville, MO). QuikChange II XL site-directed mutagenesis kits (Cat#: 200522) were from Agilent Technologies (Santa Clara, CA). TnT Quick Coupled Transcription/Translation Systems (Cat#: L1170) were from Promega (Madison, WI). Immobilized protein A/G agarose beads (Cat#: 20421) were from Pierce (Rockford, IL). All other reagents were of the highest grade available commercially.

### Cloning and mutagenesis

V5-tagged PRMT4 truncations were cloned into *pcDNA3.1D-His-V5-TOPO* plasmid using PCR-based approaches as previously described [55]. Mutagenesis was introduced by using a QuickChange II XL site-directed mutagenesis kit according to the manufacturer’s instructions. The accuracy of the mutagenesis was confirmed by sequencing. The primers used in the construction of PRMT4 truncations and site-directed mutagenesis are listed in Table 1.

**Table 1:** Primers used in the study.

Truncation	PRMT4F1	CACCATGGCAGCGGCGGCAGCG
	PRMT4F2	CACCATGCACGCGGAGCAGCAG
	PRMT4F3	CACCATGATCACCCTGGGCTGCAAC
	PRMT4F4	CACCATGTTCCAGTTCTATGGC
	PRMT4F5	CACCATGTTTTTTGCTGCTCAAGC
	PRMT4F6	CACCATGCAAGTGGACATTATC
	PRMT4F7	CACCATGGAACAGCTCTACATGG
	PRMT4R1	ACTCCCATAGTGCATGGTGTTG
	PRMT4R2	GGAGTGGGTGTGATTGACAATC
	PRMT4R3	TGTTCCACATATTCTCCGAGG
	PRMT4R4	GTCATAGCTCTGTCTTTTG
	PRMT4R5	TATGGAGCCAATGAAAGCAAC
	PRMT4R6	CATCAGGATCCGGATGTCAAATG
	DN131f	CACCATGACACTGGAGCGCTCTGTGTTC
	DN132f	CACCATGCTGGAGCGCTCTGTGTTCAG
	DN133f	CACCATGGAGCGCTCTGTGTTCAGTG
	DN134f	CACCATGCGCTCTGTGTTCAGTGAG
	DN135f	CACCATGTCTGTGTTCAGTGAGCGGAC
	DN136f	CACCATGGTGTTCAGTGAGCGGACAG
	DN137f	CACCATGTTCAGTGAGCGGACAGAGG
	DN138f	CACCATGAGTGAGCGGACAGAGGAATC
	DN105f	CACCATGAACAGCGTCCTCATCCAG
	DN110f	CACCATGCAGTTTGCCACACCCCACG
	DN115f	CACCATGCACGATTTCTGTTCTTTCTAC
	DN120f	CACCATGTTCTACAACATCCTGAAAAC
	DN130f	CACCATGCACACACTGGAGCGCTCTG
	DN140f	CACCATGCGGACAGAGGAATCCTCAG
Mutagenesis	T132Cf	CCTGTCGGGGCCACTGCCTGGAGCGCTCTGTG
	T132Cr	CACAGAGCGCTCCAGGCAGTGGCCCCGACAGG
	T132Df	CCTGTCGGGGCCACGACCTGGAGCGCTCTGTG
	T132Dr	CACAGAGCGCTCCAGGTCGTGGCCCCGACAGG
	S136Cf	CCACACACTGGAGCGCTGTGTGTTCAGTGAGCGG
	S136Cr	CCGCTCACTGAACACACAGCGCTCCAGTGTGTGG
	S136Df	CCACACACTGGAGCGCGATGTGTTCAGTGAGCGG
	S136Dr	CCGCTCACTGAACACATCGCGCTCCAGTGTGTGG
	T132AS136Af	CCACGCACTGGAGCGCGCTGTGTTCAGTGAGCGG
	T132AS136Ar	CCGCTCACTGAACACAGCGCGCTCCAGTGCGTGG
	T132CS136Df	CCACTGCCTGGAGCGCGATGTGTTCAGTGAGCGG
	T132CS136Dr	CCGCTCACTGAACACATCGCGCTCCAGGCAGTGG
	K463Af	GACCAGACAGGCTCCGCGTCCAGTAACCTGCTGG
	K463Ar	CCAGCAGGTTACTGGACGCGGAGCCTGTCTGGTC
	K471Af	GTAACCTGCTGGATCTAGCGAACCCCTTCTTCAGG
	K471Ar	CCTGAAGAAGGGGTTCGCTAGATCCAGCAGGTTAC
	K310Af	CATGGAGCAGTTCACCGCAGCCAACTTCTGGTACC
	K310Ar	GGTACCAGAAGTTGGCTGCGGTGAACTGCTCCATG
	K228Af	GCAGAGGTCCTGGTGGCGAGTAACAATCTGACAG
	K228Ar	CTGTCAGATTGTTACTCGCCACCAGGACCTCTGC
	K242Af	CGTGGTCATCCCTGGCGCAGTAGAGGAGGTCTC
	K242Ar	GAGACCTCCTCTACTGCGCCAGGGATGACCACG

The orientation of all primers is in 5′ to 3′ direction.

### Plasmid transfection

All plasmids were introduced into cells using electroporation executed with a nuclear transfection apparatus (Amaxa Biosystems, Gaithersburg, MD) with a preset program (T-013 for MLE12 and BEAS-2B cells), following the manufacturer’s instructions as previously described [55]. Briefly, one million cells in 100 μL of transfection buffer (20 mM Hepes in PBS buffer) were mixed with 3 μg of plasmids (including expression and shRNA constructs). After electroporation, the cells were cultured with 2 mL conditional medium in 6-well plates for 24 h for further analysis.

### Immunoblotting and co-IP

Immunoblotting and co-IP were conducted as previously described [55]. Briefly, for immunoblotting, whole-cell extracts (normalized to total protein concentration) were resolved by sodium dodecyl sulfate–polyacrylamide gel electrophoresis and transferred to membranes by electroblotting. The membranes were blocked with 5% (w/v) non-fat milk in Tris-buffered saline and probed with primary antibodies as indicated. Membranes were developed by an enhanced chemiluminescence (ECL) system. For IP, 1 mg of cell lysates (in PBS with 0.5% Tween 20 plus protease inhibitors) were incubated with specific primary antibodies for 2 h at room temperature. The mixture was added with 35 µL of protein A/G agarose beads for an additional 2 h at room temperature. The precipitated complex was washed for three times with 0.5% Tween 20 in PBS and analyzed by immunoblotting with ECL system.

### Real‐time PCR

Total RNA from cells was isolated with the RNeasy Mini Kit (Qiagen, Valencia, CA, USA), and reverse-transcribed by using the High-Capacity RNA-to-cDNA Kit (Applied Biosystems, Foster City, CA, USA) following the manufacturer’s manual instructions. Single-stranded cDNA was then amplified by RT‐PCR with specific primers of PRMT4 and GAPDH. RT‐PCR was performed on an ABI Prism 7000 thermocycler (Applied Biosystems, Thermo-Fisher Scientific, Waltham, MA, USA) with SYBR Green PCR Master Mix (Roche, Basel, Switzerland). For each experiment, samples (*n* = 3) were run in triplicate. Relative gene expression was calculated using the comparative CT method.

### In vitro pull-down assay

We conducted in vitro binding assays to identify the FBXO9-binding domain within PRMT4. V5-tagged PRMT4 truncated or site-directed mutant proteins were in vitro expressed using a TnT coupled reticulocyte system. Endogenous FBXO9 protein was obtained by FBXO9 IP from MLE12 cell lysate. FBXO9-precipitated protein A/G agarose beads were incubated with a variety of PRMT4 truncations or mutants for 2 h. The beads were washed extensively with 0.5% Tween 20 in PBS and analyzed by V5 and FBXO9 immunoblotting.

### Cell death detection

Lactate dehydrogenase (LDH) assay kit (Takara, Japan) was used to detect the epithelial cell death according to the manufacturer’s instructions. Absorbance of the samples at wavelength of 490 and 620 nm (background) were measured using a SpectraMax M2 microplate reader (Molecular Devices, CA, USA). The LDH activity was determined by subtracting the optical density (OD) at 620 nm absorbance value from the OD at 490 nm. The fold change of epithelial cell death was indicated with the relative LDH activity comparing to the control group and plotted in a bar graph.

### Lentivirus production

Lentiviruses were generated by Lenti-X Packaging Single Shots VSV-G (Cat#: 631276, Clontech, CA) according to the manufacturer’s procedures. Lentivirus-containing supernatants were concentrated using Lenti-X Concentrator (Cat#: 631231). Concentrated lentiviruses were resuspended in PBS and titrated by Lenti-X GoStix (Cat#: 631280). The samples were aliquoted and stored at −80 °C.

### Mouse LPS-induced lung injury procedures

All animal protocols and procedures were reviewed and approved by the University of Pittsburgh Institutional Animal Care and Use Committee (IACUC protocol #: IS00010084). LPS-induced lung injury model was conducted as previously described. Briefly, C57BL/6J mice at the age of 10 weeks were anesthetized by injecting intraperitoneally a solution of 1:1 ketamine (100 mg/kg) and xylazine (20 mg/kg) throughout the experiment. LPS (7 mg/kg) were i.t. administrated. The mice were observed for 48 h and sacrificed. Lung tissues were collected and applied for hematoxylin–eosin staining, TUNEL assay, and western blotting.

### TUNEL assay

Mouse lung tissues were fixed in 10% formalin for 48 h and then embedded in paraffin and sectioned. Slides were deparaffinized and permeabilized with 0.1% Triton X-100 in citrate buffer (0.1%, pH 6.0). TUNEL staining was performed on these slides using an In Situ Cell Death Detection Kit (Sigma-Aldrich, Cat#: 11684795910) according to the manufacturer’s instructions. The slides were visualized by a confocal microscope (NIKON A1 Spectral Confocal, Japan) with excitation wavelength in the range of 450–500 nm and detection in the range of 515–565 nm.

### Statistics

The data represent the mean ± standard deviation in the graphs depicting the error bars or as specifically indicated. Prism 7 (GraphPad software, San Diego, CA) was used to determine statistical significance. Comparisons between groups were made using unpaired, two-tailed Student’s *t* test (two groups) and one-way analysis of variance with post hoc Tukey honestly significant difference or Bonferroni and Holm multiple comparisons. Kaplan–Meier estimate was used for survival analysis in mouse septic models. *P* < 0.05 indicates statistical significance.

## Data Availability

The data that supporting the finding of this study are available from the corresponding author upon reasonable request.
